# Correction: An Empirical Biomarker-Based Calculator for Cystic Index in a Model of Autosomal Recessive Polycystic Kidney Disease—The Nieto-Narayan Formula

**DOI:** 10.1371/journal.pone.0168319

**Published:** 2016-12-09

**Authors:** The PLOS ONE Staff

## Notice of Republication

This article was republished on October 10, 2016, to correct an error where an ORCID iD was erroneously assigned to Jake A. Nieto, which was introduced during the typesetting process. The publisher apologizes for the error.
